# Near-Infrared Fluorescence Imaging of Carbonic Anhydrase IX in Athymic Mice Bearing HT-29 Tumor Xenografts

**DOI:** 10.1155/2016/6825712

**Published:** 2016-08-29

**Authors:** Jianbo Li, Baoliang Bao, Lei Liu, Xuemei Wang

**Affiliations:** Department of Nuclear Medicine, Inner Mongolia Medical University Affiliated Hospital, Hohhot 010050, China

## Abstract

Near-infrared fluorescence (NIRF) imaging technology is a highly sensitive imaging modality and has been widely used in noninvasively studying the status of receptor expression in small animal models, with an appropriate NIRF probe targeting a specific receptor. In this report, Cy5.5-conjugated anti-CAIX monoclonal antibody (Mab-Cy5.5) was evaluated in athymic mice bearing HT-29 tumor xenografts in order to investigate the effect of conjugate on tumor targeting efficacy. In vitro binding studies showed that Mab-Cy5.5 could specifically bind to the cells which expressed CAIX. Results from in vivo imaging showed that HT-29 tumor xenografts can be clearly visualized at 48 h after injection of Mab-Cy5.5, and in the blocking experiment, free anti-CAIX antibody effectively blocked the concentration of Mab-Cy5.5 in the tumors. Western blotting and immunohistochemistry analysis of HT-29 tumor xenografts verified the expression of CAIX in HT-29 tumors. Mab-Cy5.5 could specifically bind to the tumors which expressed CAIX. These results suggested that Mab-Cy5.5 was suitable for CAIX expression imaging in the preclinical research.

## 1. Introduction

Optical imaging technologies offer the distribution of a tracer in a target area, similarly to PET or SPECT, without the use of ionizing radiation or radioactive materials [1]. Near-infrared fluorescence (NIRF) with emission wavelengths between 650 and 900 nm offers improved tissue penetration and reduces autofluorescence from nontarget tissues [2]. NIRF imaging technology is a highly sensitive imaging modality and has been widely used in noninvasively studying the status of receptor expression in small animal models, with an appropriate NIRF probe targeting a specific receptor [1, 3]. Near-infrared fluorescence imaging probe (NIR760-XLP6) could preferentially bind to the type 2 cannabinoid receptors (CB_2_R) over the type 1 cannabinoid receptors in in vitro binding test. Furthermore, NIR760-XLP6 showed obviously uptake in mouse tumor models DBT-CB2, which expressed CB_2_R [4]. For the cell adhesion molecule integrin *α*_v_ *β*_3_, NIRF probes Cy5.5-RGD peptides (monomer, dimer, and tetramer) would allow NIRF imaging of mice bearing U87MG tumor xenografts [5].

Carbonic anhydrase IX (CAIX) is one of the tumor-associated proteins, frequently overexpressed in a broad range of hypoxic tumors and absent from their normal counterparts [6–9]. It is thought to be playing a role in the regulation of cell proliferation, cell adhesion, and tumor progression [10–13]. Because of being overexpressed in hypoxic tumors with limited distribution in normal tissues, CAIX is clearly an attractive target for diagnostic imaging and also as a potential biomarker of treatment response [14, 15].

Monoclonal antibody (Mab), which is shown to bind to an epitope of some antigen, has received extensive attention in the research of tumor diagnosis and therapy applications [16]. In the present study, we focused on a new anti-CAIX monoclonal antibody, studying the specific optical imaging of CAIX in the mice bearing human HT-29 tumor xenografts using Cy5.5-conjugated anti-CAIX monoclonal antibody (Mab-Cy5.5). NIRF imaging technology was used to characterize the presence of CAIX at the molecular level.

## 2. Material and Methods

### 2.1. General

Mouse anti-human CAIX monoclonal antibody was purchased from R&D Systems, Inc. (Minneapolis, MN, USA). Goat anti-human CAIX polyclonal antibody was purchased from Santa Cruz Biotechnology, Inc. (Dallas, TX, USA).

HRP-conjugated rabbit anti-goat IgG was purchased from Jackson ImmunoResearch Europe Ltd. (Newmarket, UK). Mono-Reactive NHS Ester of Cy5.5 dye (Cy5.5-NHS) and PD-10 columns were purchased from GE HealthCare (Fairfield, CT, USA). 10% Bis-Tris Gel for SDS-PAGE was purchased from Thermo Fisher Scientific Inc. (Waltham, MA, USA). BeyoECL Plus Kit was purchased from Beyotime Institute of Biotechnology (Shanghai, China). Multiwell glass bottom culture plates were purchased from MatTek Corporation (Ashland, MA, USA).

### 2.2. Synthesis and Characterization of Mab-Cy5.5

About 0.9 mg anti-CAIX Mab (0.017 umol, MW 53 kDa) was dissolved in 1900 *μ*L conjugation buffer (0.1 M Na_2_CO_3_/NaHCO_3_, pH 9.5) and then mixed with 0.9 mg Cy5.5-NHS (1.26 umol, MW 716.31) in 90 *μ*L anhydrous DMSO. The molar ratio of Cy5.5-NHS to anti-CAIX Mab was about 74. After the reaction being at room temperature (RT) for 45 min, the crude product was passed through PD-10 column and eluted with PBS. The Mab-Cy5.5 concentration and Cy5.5/Mab ratio were measured by UV spectrophotometer (HITACHI U-3010, Hitachi, Ltd., Tokyo, Japan).

### 2.3. Cell Culture

Hela (human cervical cancer cell line), HT-29 (human colon cancer cell line), and PANC-1 (human pancreatic cancer cell line) were all obtained from Cell Bank of Chinese Academy of Sciences (Shanghai, China). RCC4 (human renal cell carcinoma cell line) was purchased from Sigma-Aldrich Co. (St. Louis, MO, USA). HT-29 was cultured in McCoy's 5A medium with 10% new-born calf serum (NBCS). Hela, PANC-1, and RCC4 were cultured in Dulbecco's modified Eagle medium (DMEM) with 10% NBCS. All cells were maintained in a humidified atmosphere containing 5% CO_2_ at 37°C. For the hypoxic treatments, the cells were placed in an anaerobic incubator with 1% O_2_, 5% CO_2_, and 94% N_2_. McCoy's 5A medium, DMEM, NBCS, and PBS (pH 7.4) were purchased from Thermo Fisher Scientific Inc. (Waltham, MA, USA).

### 2.4. In Vitro Binding Study

About 1 × 10^4^ Hela cells were seeded in 24-well glass bottom culture plates for 24 h at 37°C. For the hypoxic treatment, cells were exposed to hypoxia for another 24 h. Then cells were washed twice with PBS and incubated with 0.5 mL culture medium with Mab-Cy5.5 (10 nM) for 1 h at 37°C. After the incubation, the cells received the examination of fluorescence microscopy. The fluorescence signals of the cells were imaged with an AxioSkop fluorescence microscope (ZEISS) equipped with Cy5.5 filter set and a charge-coupled camera (AxioCam MRC5, ZEISS). HBO 100 W microscopic illuminator was used as a light source for fluorescence excitation. HT-29, PANC-1, and RCC4 cells were taken by the same operations as Hela cells.

RCC4 cells with the treatment of hypoxia or without were incubated with 0.25 mL culture medium with free Mab (100 nM) and 0.25 mL culture medium with Mab-Cy5.5 (10 nM) for 1 h at 37°C for the competition study. RCC4 cells with the treatment of hypoxia or without were incubated with 0.5 mL culture medium with free Cy5.5 (100 nM) for 1 h at 37°C in order to determine whether nonspecific binding between Cy5.5 and the cells would occur.

In in vitro binding study and competition study, all cells were washed three times with 0.5 mL PBS after the incubation and then received the examination of fluorescence microscopy.

### 2.5. Tumor Xenografts

About 5 × 10^6^ HT-29 cells suspended in 100 *μ*L PBS were subcutaneously injected into the right flank of athymic female mice (4–6 weeks old). The mice were subjected to in vivo imaging studies when the tumors reached ~0.6 cm in diameter. The animal study proposal had been approved by the Medical Ethics Committee of Inner Mongolia Medical University. The animal experiments were performed in accordance with the “Guide for the Care and Use of Laboratory Animals” (Institute of Laboratory Animal Resources, Commission on Life Sciences, National Research Council, ISBN: 0-309-58869-3, 140 pages, 1996).

### 2.6. In Vivo Fluorescence Imaging

The IVIS Lumina II used for in vivo fluorescence imaging was from Caliper Life Sciences, Inc. (Waltham, MA, USA). Images were acquired using Living Image software. The tumor-bearing mice in the experiment (*n* = 3) received 0.25 nmol Mab-Cy5.5 via the tail vein and were subjected to optical imaging at various time points postinjection (p.i.). For the blocking experiment, the other tumor-bearing mice (*n* = 3) were injected with 1.5 nmol free Mab 24 h before the injection of 0.25 nmol Mab-Cy5.5 via the tail vein. All NIRF images were acquired using 30 s exposure time (*f*/stop = 2). The mouse in the experiment was sacrificed after in vivo fluorescence imaging. The tumor and major tissues and organs were dissected for fluorescence imaging.

### 2.7. Western Blotting and Immunohistochemistry Analysis

Tumors were cut into pieces and lysed in RIPA buffer containing 1 mM PMSF. Samples containing 40 *μ*g proteins were separated on a 10% Bis-Tris gel by SDS-PAGE and then transferred to PVDF membrane. The membrane was incubated with goat anti-human CAIX polyclonal antibody (1 : 200) for 2 h at RT and then incubated with HRP-conjugated rabbit anti-goat IgG (1 : 1000) for 1 h at RT. Membrane-bound secondary antibodies were detected by BeyoECL Plus Kit.

Experimental procedure of immunohistochemistry was seen in our previous report [17]. 

## 3. Results

### 3.1. Conjugation and Purification of Mab-Cy5.5

**Scheme 1:**
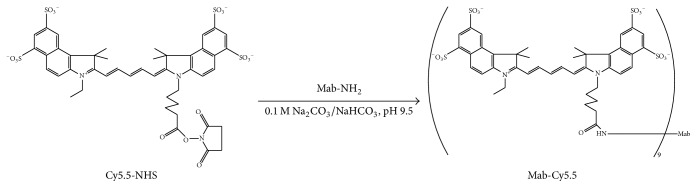
Reaction scheme for the synthesis of Mab-Cy5.5.

The synthesis of Mab-Cy5.5 was achieved through conjugation of Cy5.5-NHS ester with free amino groups of Mab (Scheme 1). The desired products were purified by PD-10 column. Cy5.5/Mab ratio, measured by UV spectrophotometer, was 9. Mab-Cy5.5 was diluted with PBS for in vivo and in vitro use.

### 3.2. In Vitro Binding Characteristics

**Figure 1:**
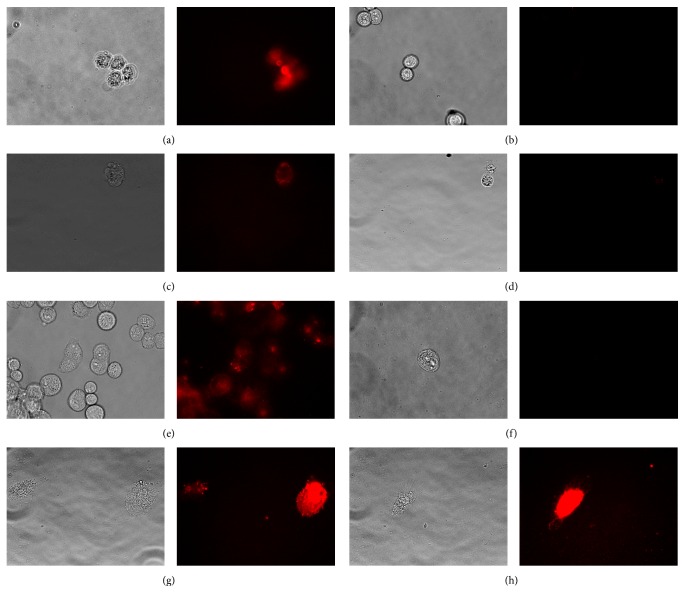
In vitro binding characteristics of Mab-Cy5.5. NIRF images were obtained after Hela, HT-29, PANC-1, and RCC4 cells under hypoxia (a, c, e, and g) or under normoxia (b, d, f, and h) were incubated with 10 nM Mab-Cy5.5.

To demonstrate binding characteristics of Mab-Cy5.5, hypoxic and normoxic Hela, HT-29, PANC-1, and RCC4 cells were incubated with Mab-Cy5.5. From Figures 1(a), 1(c), and 1(e), we could observe that Hela, HT-29, and PANC-1 cells with the treatment of hypoxia all emitted fluorescence signal, but for the corresponding cells (Figures 1(b), 1(d), and 1(f)) without the treatment of hypoxia, there was no appearance of fluorescence signal. In Figures 1(g) and 1(h), regardless of RCC4 cells in hypoxic condition or in normoxic condition, fluorescence signal could be detected.

**Figure 2:**
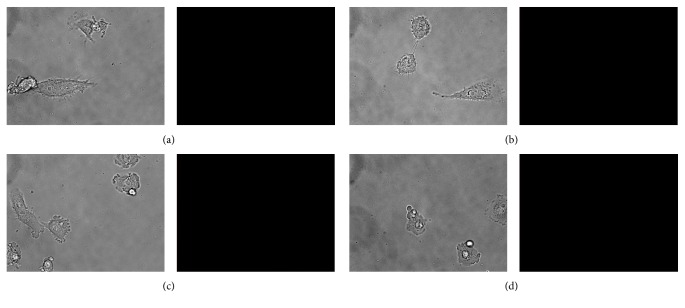
In vitro binding characteristics of Mab-Cy5.5. RCC4 cells were incubated with (a) 10 nM Mab-Cy5.5 and 100 nM free Mab under hypoxia; (b) 10 nM Mab-Cy5.5 and 100 nM free Mab under normoxia; (c) 100 nM free Cy5.5 under hypoxia; (d) 100 nM free Cy5.5 under normoxia.

NIRF images (Figures 2(a) and 2(b)) showed that there was no appearance of fluorescence signal in RCC4 cells with the treatment of hypoxia or without. It was observed from Figures 2(c) and 2(d) that hypoxic or normoxic RCC4 cells with incubation with free Cy5.5 showed no appearance of fluorescence signal, suggesting that there was no nonspecific binding between Cy5.5 and RCC4 cells.

### 3.3. In Vivo Fluorescence Imaging

**Figure 3:**
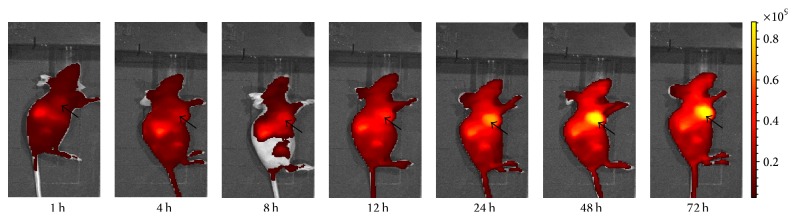
In vivo fluorescence imaging of athymic mouse bearing HT-29 tumor xenografts at 1–72 h after injection of 0.25 nmol Mab-Cy5.5. The location of the tumor was indicated by an arrow.

Athymic mice bearing HT-29 tumor xenografts in the experiment received 0.25 nmol Mab-Cy5.5 via the tail vein and were subjected to fluorescence imaging at various time points p.i. NIRF images from Figure 3 showed that a little accumulation of fluorescence signal (Radiant Efficiency = 2.4 × 10^8^ (p/sec/cm^2^/sr)/(*μ*W/cm^2^)) was found in the tumor at 1 h p.i., but until 48 h p.i., there was obvious fluorescence signal in the tumor, which reached the highest level (Radiant Efficiency = 9.18 × 10^8^ (p/sec/cm^2^/sr)/(*μ*W/cm^2^)) compared to fluorescence signal in the tumor at other time points p.i. From 1 h p.i. to 72 h p.i., fluorescence signal in the liver and kidney was gradually increased.

**Figure 4:**
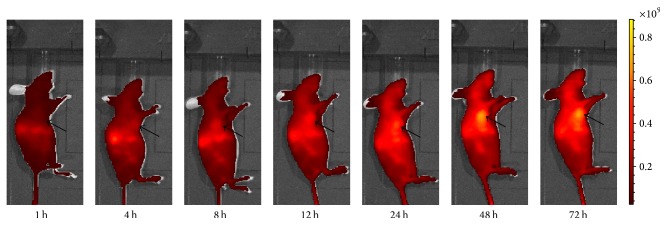
In vivo fluorescence imaging of athymic mouse bearing HT-29 tumor xenografts at 1–72 h after injection of 0.25 nmol Mab-Cy5.5 and 1.5 nmol free Mab. The location of the tumor was indicated by an arrow.

Athymic mice bearing HT-29 tumor xenografts in the blocking experiment received 0.25 nmol Mab-Cy5.5 and 1.5 nmol free Mab via the tail vein and were subjected to fluorescence imaging at various time points p.i. Compared to Figure 3, fluorescence signal in the tumor from Figure 4 at various time points p.i. was greatly reduced.

**Figure 5:**
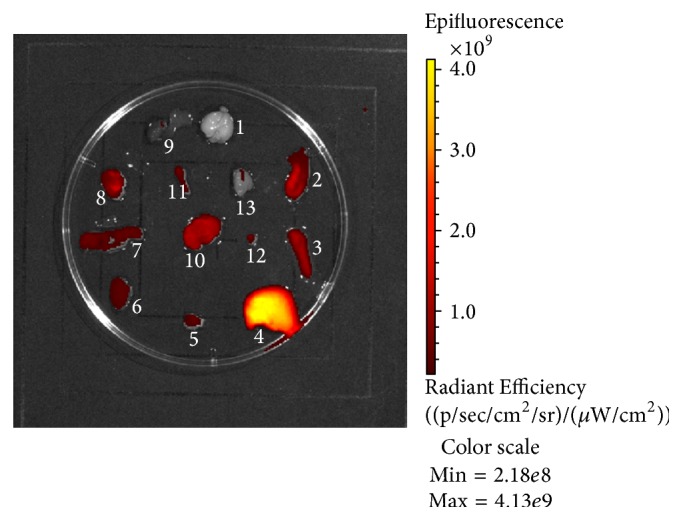
Representative image of dissected organs of mouse in the experiment sacrificed at 72 h after injection of Mab-Cy5.5 (1, brain; 2, small intestine; 3, spleen; 4, liver; 5, lung; 6, kidney; 7, cecum; 8, stomach; 9, heart; 10, tumor; 11, bone; 12, bladder; 13, muscle).

NIRF image of dissected organs of mouse in the experiment was shown in Figure 5. There were strong fluorescence signal in liver (Radiant Efficiency = 16.6 × 10^8^ (p/sec/cm^2^/sr)/(*μ*W/cm^2^)) and medium fluorescence signal in tumor (Radiant Efficiency = 8.37 × 10^8^ (p/sec/cm^2^/sr)/(*μ*W/cm^2^)) compared to the other organs. Medium fluorescence signal was also found in small intestine (Radiant Efficiency = 4.46 × 10^8^ (p/sec/cm^2^/sr)/(*μ*W/cm^2^)) and stomach (Radiant Efficiency = 3.01 × 10^8^ (p/sec/cm^2^/sr)/(*μ*W/cm^2^)).

### 3.4. Western Blotting and Immunohistochemistry Analysis

**Figure 6:**
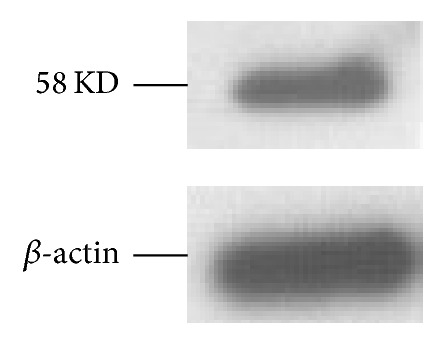
Western blotting analysis of HT-29 tumor xenografts. *β*-actin was included as a loading control.

**Figure 7:**
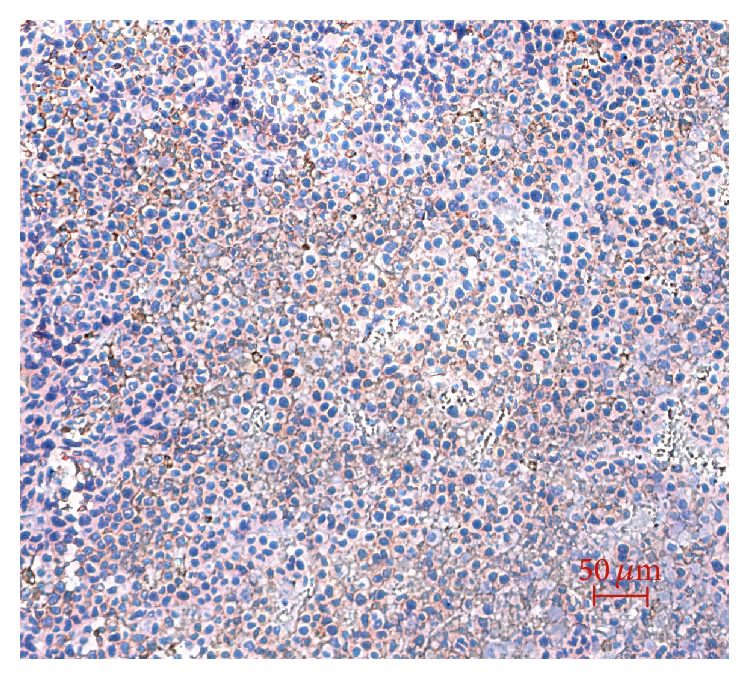
Immunohistochemical analysis of HT-29 tumor xenografts. Scale bar: 50 *μ*m.

To further characterize the expression of CAIX in HT-29 tumor xenografts, the tumor of the tumor-bearing mouse was removed for research. Western blotting analysis revealed the presence of CAIX specific 58 KD band in Figure 6. As expected, CAIX staining clearly appeared in the tumor and at the plasma membrane of the tumor cells from immunohistochemical analysis in Figure 7.

## 4. Discussion

NIRF image technology, which uses neither ionizing radiation nor radioactive materials, is a valuable tool for noninvasively studying the status of receptor expression in small animals. It uses a sensitive camera to detect fluorescence emission from fluorophores in whole-body living small animals [1, 3]. In this paper, we described the use of Mab-Cy5.5 to image CAIX in athymic mice bearing human HT-29 tumor xenografts. The conjugates were conveniently synthesized from the free amino groups of Mab and Cy5.5-NHS ester (Scheme 1).

Specific binding of Mab-Cy5.5 to Hela, HT-29, and PANC-1 cells with the treatment of hypoxia occurred, but it did not happen to Hela, HT-29, and PANC-1 cells without the treatment of hypoxia (Figures 1(a)–1(f)). Hela, HT-29, and PANC-1 cells only express CAIX in hypoxic condition but they do not express CAIX in normoxic condition [18, 19]. RCC4 cells with VHL gene inactivation could constitutively express CAIX all the time regardless of the treatment of hypoxia [9, 20]. From Figures 1(g) and 1(h), we found that there was the appearance of fluorescence signal in both images, suggesting that Mab-Cy5.5 could bind to RCC4 cells with the treatment of hypoxia or without. In the blocking experiment of in vitro binding, NIRF images (Figures 2(a) and 2(b)) showed that there was no appearance of fluorescence signal in RCC4 cells with the treatment of hypoxia or without, indicating that free Mab could effectively block the binding of Mab-Cy5.5 to RCC4 cells, and binding of Mab-Cy5.5 to RCC4 cells was mediated through CAIX. Mab-Cy5.5 could bind to the cells which expressed CAIX (Figures 1 and 2). The possibility of nonspecific binding between the cells and free Cy5.5 was excluded by our findings (Figures 2(c) and 2(d)) that no fluorescence signal was detected in RCC4 cells incubated with Cy5.5.

In in vivo fluorescence imaging experiment, accumulation of Mab-Cy5.5 in HT-29 tumor xenografts was clearly seen (Figure 3). A little accumulation of Mab-Cy5.5 was found in the tumors at 1 h p.i., but until 48 h p.i., there was obvious fluorescence signal in the tumors compared to the other normal organs or tissues. In our other research, ^125^I-labeled Mab also preferentially accumulated in the xenografted HT-29 tumors at 48 h p.i. [17]. It was speculated that Mab could have early uptake in the tumors due to the tumor vasculature being permeable to such large molecule, and with time passing, Mab was diffusing away from the tissues adjacent to the vasculature and moved to the poorly perfused hypoxic regions rich in CAIX. Furthermore, the uptake of Mab-Cy5.5 in HT-29 tumor xenografts was blocked by the free Mab in Figure 4. Compared to Figure 3, fluorescence signal from Figure 4 at various time points p.i. was greatly reduced. Therefore, both in vitro and in vivo studies supported that Mab-Cy5.5 specifically targeted CAIX.

Fluorescence signal in the liver and kidney from 1 h to 72 h p.i. was gradually increased (Figure 3), suggesting that Mab-Cy5.5 was primarily cleared through the hepatobiliary metabolism and urinary system. Consistent with our in vivo fluorescence imaging findings, there were strong fluorescence signal in liver and medium fluorescence signal in tumor compared to the other organs listed (Figure 5). Small intestine and stomach are known to express CAIX [21, 22], which would explain the medium level of small intestine and stomach uptake of Mab-Cy5.5 in Figure 5. Western blotting and immunohistochemical analysis were both used to testify the expression of CAIX in HT-29 tumor xenografts (Figures 6 and 7).

## 5. Conclusions

In our study, Mab-Cy5.5 allowed NIRF imaging of HT-29 tumor xenografts in mice, providing noninvasive means for the detection and characterization of CAIX-expressing tissues without the harmful effect of radiation rays (such as PET or SPECT). Mab-Cy5.5 was suitable for CAIX expression imaging in athymic mice bearing HT-29 tumor xenografts.
